# i2b2-ML: module to facilitate machine learning in the informatics for integrating biology and the bedside platform

**DOI:** 10.1093/jamiaopen/ooag047

**Published:** 2026-05-25

**Authors:** Jeffery G Klann, Micheal M Mendis, Shyam Visweswaran, Shawn N Murphy, Hossein Estiri, Kavishwar B Wagholikar

**Affiliations:** Laboratory of Computer Science, Department of Medicine, Massachusetts General Hospital, Boston, MA, United States; MassGeneral Brigham, Somerville, MA, United States; Department of Biomedical Informatics, University of Pittsburgh, PA, United States; MassGeneral Brigham, Somerville, MA, United States; Laboratory of Computer Science, Department of Medicine, Massachusetts General Hospital, Boston, MA, United States; Laboratory of Computer Science, Department of Medicine, Massachusetts General Hospital, Boston, MA, United States

**Keywords:** data science, data platform, software, open-source, reproducibility

## Abstract

**Objective:**

Although machine learning (ML) holds significant potential to transform healthcare, there has been a recent surge in research output that often lacks methodological rigor, contributing to a reproducibility crisis. Additionally, the growing reliance on electronic health records (EHR) for developing ML models has heightened concerns about patient data privacy. To tackle these challenges, we have extended the open-source i2b2 (Informatics for Integrating Biology and the Bedside) platform to allow researchers to train and run ML models without requiring manual programming or direct access to patient-level data.

**Materials and Methods:**

We have developed a proof-of-concept ML module for the i2b2 platform for creating and executing ML models. We describe the design of the module and demonstrate its use on a publicly available Kaggle dataset. Next we test its scalability on a large real-world dataset.

**Results:**

Model training with EHR of 100,000 patients randomly selected from the MIMIC-IV dataset was completed in 75.8 minutes and the developed model was applied to classify 28,985 patients in 1.61 minutes.

**Discussion:**

Implementation of the ML functionalities of the i2b2-ML module was successfully evaluated with a publicly available dataset. The developed module allows seamless training and execution of ML models without the need for manual programming and export of patient-level data, thus addressing many of the challenges associated with data privacy and reproducibility.

**Conclusion:**

In summary, the developed i2b2-ML module can reduce the technical overhead for researchers for applying ML to health data. Future work will focus on improving the i2b2 graphical interface to further simplify the use of the ML module and on streamlining the distribution of the ML module to existing i2b2 installations, so researchers can more easily analyze EHR data that exists in their current installations.

## Introduction

Machine learning (ML) offers many potentials to transform different aspects of healthcare by enabling predictive models, personalized treatments, and improved patient outcomes.^1^ However, the rapid growth of research in this area has been accompanied by a lack of methodological rigor, making it difficult to reproduce findings and undermining trust in the field.^2–4^ At the same time, the widespread use of electronic health records (EHR) as a primary data source for ML models has intensified scrutiny over patient privacy and data security, as large volumes of protected health information are being increasingly used for ML.^5^^,^^6^ To tackle these challenges, we have extended the i2b2 (Informatics for Integrating Biology and the Bedside) platform, ^7^ to develop the i2b2-ML module that allows researchers to train and run ML models without requiring manual programming or direct access to patient-level data.

The i2b2 platform has been widely used in academic medical centers and consortia to enable the integration and querying of diverse healthcare data from multiple sources.^7^^,^^8^ Researchers use i2b2 to query large repositories of clinical data, which may include diagnosis, laboratory, medication, billing information, clinical notes, radiographic images, and other patient-related data.^9^ The platform offers an intuitive interface, enabling researchers to build complex queries without requiring programming expertise, to find patients who meet specific inclusion and exclusion criteria. For instance, a researcher might want to study the effectiveness of a diabetes medication. Using i2b2, they can define the cohort-query to select patients based on criteria like demographics, age, diagnosis of diabetes or a laboratory result for blood glucose or HbA1c. Once the cohort is defined in i2b2, the platform seamlessly queries the data and compiles a list of patients who match the specified criteria. The ability to precisely define cohorts based on detailed criteria is the most utilized feature of i2b2.

After a cohort is created, researchers can download the data for more detailed analyses. This data is typically exported in CSV or Excel format and can be readily imported into statistical software or other analytical tools. The downloaded data includes patient-level data in the defined cohort, and researchers can choose which data elements are to be included in the exported file such as demographic information, treatment outcomes, and laboratory test results. Researchers can use this data export to develop analytic models to further refine the cohort or to predict individual patient outcomes.

While the i2b2 platform significantly streamlines the process of identifying and exporting patient cohorts for research, there are several challenges associated with the development of ML models external to the i2b2 environment (see Appendix A, available as Supplementary material is available at *[JAMIA Open]* online), including ensuring patient data privacy,^5^ data management,^10^ expertise.^11^ and model life-cycle management.^2^^,^^11^ Addressing these challenges often requires interdisciplinary collaboration, robust infrastructure, and adherence to best practices in data governance, ML development, and systems integration. With this motivation, we have enhanced i2b2 to support in-platform ML model development and execution, which will reduce the barriers to implementing advanced data analytics in healthcare research.

## Methods

We implemented an application programming interface (API) for the i2b2-ML platform to facilitate the creation and execution/application of ML models. The APIs allow the end-users to develop ML models within the i2b2 platform without the need to download data into external environments. The models are stored in i2b2’s database and can be invoked, with the resultant output being seamlessly integrated back into the i2b2 database.

**Figure 1.**
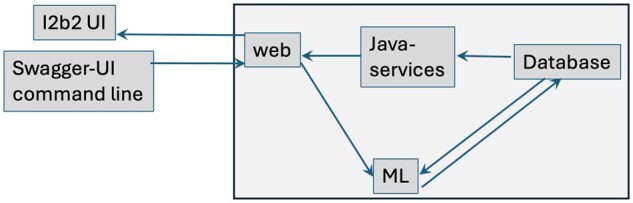
Components for implementing the ML functionality in the i2b2 platform include docker containers for webservices, java services, database, and ML. The workflow for ML involves the query interface of i2b2 for defining patient-sets, and then using the Swagger or command-line interface which accepts JSON text to trigger the processes to build or execute the models.

We implemented the module in Python by extending the i2b2-etl package that we have published previously (see Figure 1).^12^ The source code is available publicly in GitHub (https://github.com/i2b2/i2b2-etl/releases/tag/v4.1.0). We have also packaged the code into containers published in public repositories in DockerHub (https://hub.docker.com/repository/docker/i2b2/i2b2-etl/). A video demonstration is available at YouTube link and the API can be accessed at http://i2b2ml.org/i2b2ml/ (username: demo, login: Etl@2021) and http://i2b2ml.org/swagger/ (username: demo\demo, login: Etl@2021). In the following sections, we describe the API and then demonstrate the application using a publicly available dataset.

### User workflow for building and executing ML models

For developing an ML model, the researcher first creates training data by creating a positive patient-set and a negative patient-set using the i2b2 query interface. The data for the patients in these patient sets constitute the training data for creation of the ML model. The positive patient set consists of patients that have the attribute or outcome to be predicted. In contrast the negative patient set is the control group of patients that do not have the attribute or outcome of interest. The workflow for ML involves the end-user using the query interface of i2b2 for defining patient-sets, and then using the Swagger interface which accepts JSON text to trigger the processes to build or execute the models. For instance, a researcher who wants to train an ML model (e.g., for finding diabetes patients) performs the following steps (detailed in Appendix B, available as Supplementary material is available at *[JAMIA Open]* online).

**Figure 2.**
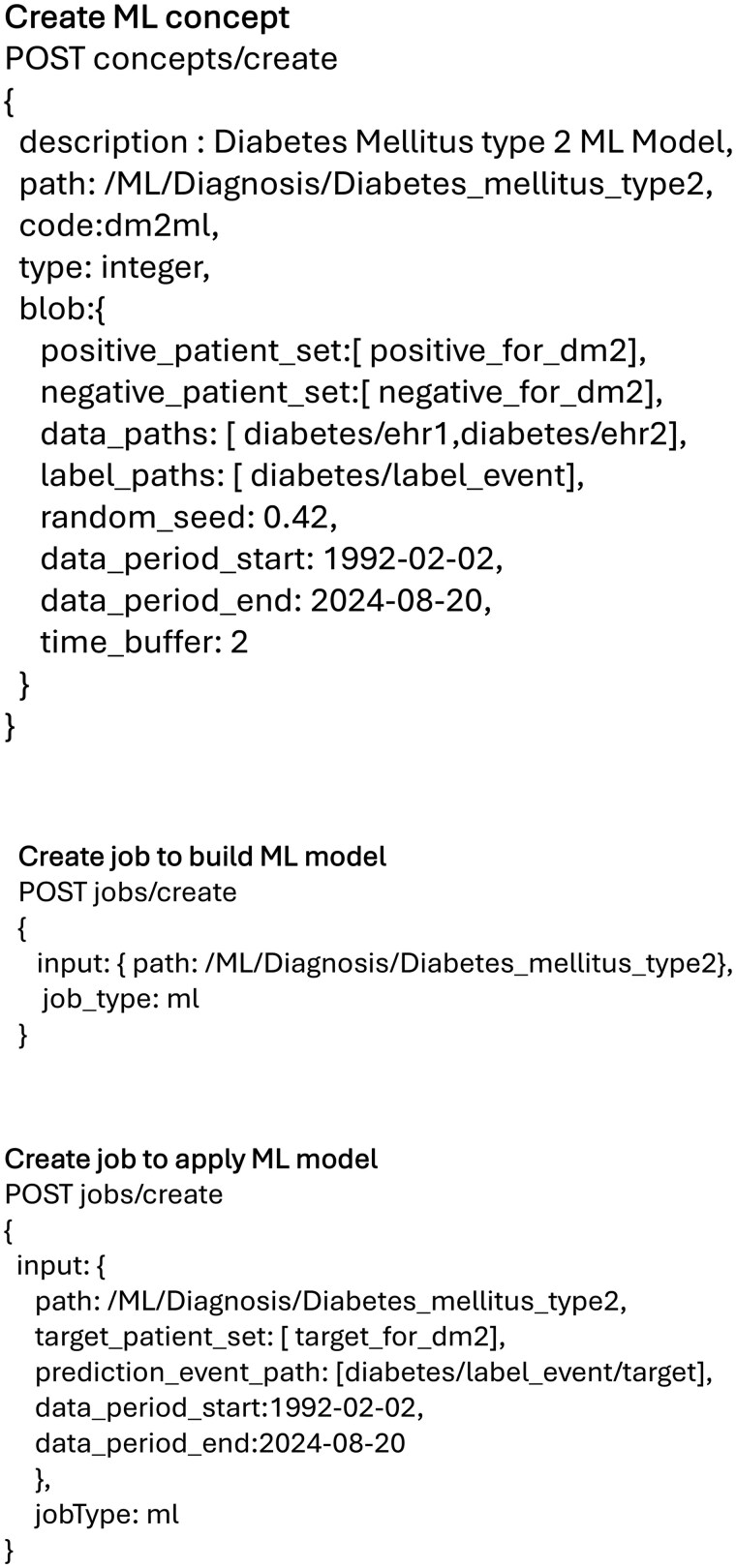
Payloads for creating the concept for the ML model and to trigger the model development respectively, showing the example for Diabetes. The quotes from JSON payloads are removed for readability.

1. The researcher creates a query in the i2b2 interface, to find patients that have been annotated for the presence of diabetes (referred as the positive set) and similarly creates a negative-set query to find patients that have been annotated for absence of diabetes.
2. After the patient sets have been created, the researcher creates a concept for the ML model with specifications in the Swagger interface. The specification for creating a diabetes model is shown in Figure 2a.
3. The researcher then triggers a process to build the ML model indicating the path to the concept where the ML model was created in the previous step. The example specifications for the diabetes example are shown in Figure 2b.

For executing the model, the researcher creates a query in the i2b2 interface, to build a ‘target patient-set’, on which the ML model will be executed on. This set could include patients that have a predisposing risk factor for the disease. Next the user proceeds to build the ML model in the Swagger interface by using the following specifications. See Figure 2c for the diabetes example.

### Datasets

We used two datasets to test the developed module.—the Kaggle Pima Indians Diabetes Database,^13^ and MIMIC-IV dataset.^14^ The Kaggle dataset contains 768 records from women of Pima Indian heritage aged ≥21, with eight numeric clinical measurements—pregnancies, 2-hr plasma glucose, diastolic blood pressure, triceps skin-fold thickness, 2-hr serum insulin, body mass index, diabetes-pedigree function, and age—plus a binary outcome. It’s widely used to build and benchmark models that predict diabetes. The MIMIC-IV dataset is a large, freely accessible, de-identified electronic health record dataset from Beth Israel Deaconess Medical Center. It contains data for 364,627 patients, of whom 223,452 had at least one hospitalization and 141,175 were seen only in the emergency department. It is widely used for ML research with detailed demographics, vitals, labs, medications, procedures, diagnoses (ICD-9/10), and outcome. MIMIC-IV is available to credentialed users via PhysioNet under a data-use agreement.

### Demonstration

We deployed the i2b2 platform and the developed ML module on an aws-EC2 instance and loaded a publicly available Kaggle dataset into the platform.^13^ Specifically, we transformed the dataset into the concept and fact CSV format required by the i2b2-etl module. We then imported the data using the i2b2-etl GUI, and then created positive and negative sets of patients using the i2b2 query tool GUI. Next, we invoked the swagger-UI using the calls shown in Figure 2, to first build the model and then apply it to a target set of patients. We verified that the predictions computed by the model were available in the platform through the i2b2 interface. Next, we tested the scalability of the tool by repeating the above steps on a large dataset—MIMIC-IV which includes data for 364,627 patients. ^14^ We performed these evaluations on Amazon EC2 g6.12xlarge instance having 48 vCPUs, and 192 GiB of memory, and 800 GB diskspace.

## Results and discussion

The ML module allows for building and execution of ML models in the platform, without the need to export patient level-data. The ML module efficiently handled both training and prediction tasks. For the Kaggle dataset, the model was trained using data from 768 patients in 1.32 s, and applied to classify 768 patients in 2.2 s. For the MIMIC-dataset, the model was trained on 100,000 patients in 75.8 min and then applied to classify 28,985 patients in 1.61 minutes. These results demonstrate the functionality and scalability of the i2b2-ML module.

### Security and privacy

The data security was significantly improved, as the entire workflow—from data extraction to ML model application—remained within the i2b2 environment, minimizing risks associated with patient data export. All models, datasets, and training parameters were stored in a traceable manner within the platform, facilitating auditability. The primary advantage of this solution is the ability to conduct advanced analytics without the need to export sensitive patient data. By eliminating data transfers, the solution reduces risks associated with data breaches and simplifies compliance with data governance regulations such as HIPAA.

### Reproducibility

**Figure 3.**
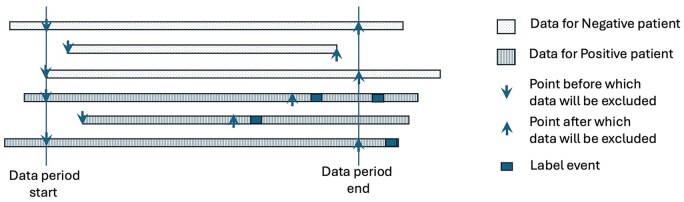
Data for patients will be excluded before the start of the data-period and after the end of data-period. Next if the label event is present in the included data, data will be excluded in the time-buffer period before the label event. The buffer period for data isolation is often necessary to avoid data leakage.

The module fosters reproducibility of analysis by ensuring that all aspects of the analytical workflow—data extraction, data-cleaning, data-isolation, feature engineering, feature selection, model training, and predictions—are performed systematically and automatically (without manual programming) within the same environment (see Figure 3). Furthermore, the API specification emphasizes data isolation, i.e., removal of data points close to the label-events, and ensuring that there is no overlap between labels, which is critical for preventing data leakage.^15^ By providing a systematized workflow, the module minimizes variability in data preprocessing and model parameters, which are familiar sources of irreproducibility. Automated analysis execution ensures that other researchers can replicate findings by accessing the same data and algorithms stored in the platform. Additionally, by storing the models directly within the i2b2 platform, the risk of errors due to platform shifts or data handling is reduced, further enhancing the reliability and reproducibility of research outcomes.

### Good data practices

The design of the ML module fosters good data practices by providing an elaborate and systematic workflow for model development and execution. By systematizing the steps required to build, train, and apply models, the framework promotes consistency, minimizes errors due to manual programming, reduces the likelihood of data leakage, and improves reproducibility. Additionally, the framework identifies bottlenecks that may hinder model performance, such as feature engineering, providing insights for further optimization. The specifications for model creation and execution outlined in the paper have utility beyond i2b2, with potential for broader application to other datasets and use cases.

### Model repository

Storing the trained models within the platform enables the i2b2 platform to serve not only as a data warehouse but also as a repository of algorithms for processing and analyzing healthcare data.^16^ Researchers can store and access a variety of ML models directly within the platform, promoting reuse and collaboration across studies. This will allow models to be shared among teams, ensuring that well-performing algorithms are accessible for future projects. Over time, the accumulation of diverse models within the platform can create a valuable library of algorithms that enhance the research ecosystem.^17–19^

### Housekeeping

The developed pipeline performs essential housekeeping tasks to ensure the smooth operation of the ML workflows. These tasks include managing extracting relevant patient data, storing the models, and cleaning temporary files. Automated housekeeping routines reduce the burden on researchers. These housekeeping functions contribute to a seamless user experience and sustainable operation of the platform.

The i2b2-ML module is designed to assist researchers who want to build and apply ML models within the i2b2 platform itself, requiring little to no programming expertise and ensuring that all data stays securely inside i2b2 without patient-level exports. In contrast, the OHDSI ‘PatientLevelPrediction’ (PLP) framework ^20^ is a powerful R-based toolkit that supports a much wider range of algorithms and extensive configurability for studies with OMOP-CDM databases, but demands significant technical expertise in R, OMOP conventions, and ML workflows. Essentially, i2b2-ML prioritizes simplicity, privacy, and ease of use within an i2b2 environment.

The i2b2-ML platform allows users not only to work with public example datasets but also to upload their own datasets. Moreover, the module is designed to integrate with existing i2b2 installations, which will allow the researchers to analyze data existing in their institutional i2b2 platform without exporting any sensitive information, supporting reproducible research, regulatory compliance, and practical ML applications tailored to their local priorities.

## Limitations and Future Work

There are several limitations to our work. Currently, i2b2-ML query interface only partially supports the end-to-end workflow for model development and execution, requiring researchers to rely on the Swagger or command-line interface to build and apply the ML models. This may pose usability challenges for researchers unfamiliar with JSON and Swagger-like interfaces. Future work will involve modifying the i2b2 query interface to incorporate functionality to train the ML models and to trigger their training and execution.

Another limitation is that the current implementation supports only logistic regression models, which limits the scope of analytics that can be performed. However, the system is designed with a modular architecture, and the ML functionality can be extended by modifying the source code to incorporate additional algorithms such as deep learning architectures and large language models.

Another key limitation is the lack of extensive testing for scalability; while the module has performed well with a large dataset, further testing is required to ensure it can handle concurrent model development tasks effectively in high-demand environments. Addressing these limitations will be crucial to enhancing the module’s usability, flexibility, and robustness in real-world settings.

## Conclusion

The developed module allows seamless training and application of ML models without the need for manual programming or access to patient-level data, thus addressing many of the challenges associated with data privacy, reproducibility, and manual effort. The module can potentially reduce the technical overhead for researchers to apply ML to health data. Future work will focus on improving the i2b2 graphical interface to further simplify the use of the ML module and on making it easier to deploy the module within existing i2b2 installations

## Supplementary Material

ooag047_Supplementary_Data

## Data Availability

The source code is available publicly in GitHub (https://github.com/i2b2/i2b2-etl/releases/tag/v4.1.0), and DockerHub (https://hub.docker.com/repository/docker/i2b2/i2b2-etl/). The Pima India dataset is available at https://www.kaggle.com/datasets/uciml/pima-indians-diabetes-database and the MIMIC-IV dataset is available at https://physionet.org/content/mimiciv/3.1/ after completing the required data use agreement.

## References

[1] Matheny ME , Whicher D, Israni ST. Artificial intelligence in health care. JAMA. 2020;323:509–510. 10.1001/jama.2019.21579 31845963

[2] Gibney E. Could machine learning fuel a reproducibility crisis in science? Nature. 2022;608:250–251. 10.1038/d41586-022-02035-w 35883008

[3] Ball P. Is AI leading to a reproducibility crisis in science? Nature. 2023;624:22–25. 10.1038/d41586-023-03817-6

[4] Ciobanu-Caraus O , Aicher A, Kernbach JM, et al A critical moment in machine learning in medicine: on reproducible and interpretable learning. Acta Neurochir (Wien). 2024;166:14. 10.1007/s00701-024-05892-8 38227273 PMC10791964

[5] Price WN , Cohen IG. Privacy in the age of medical big data. Nat Med. 2019;25:37–43. 10.1038/s41591-018-0272-7 30617331 PMC6376961

[6] Kaplow K , et al Data professionals’ attitudes on data privacy, sharing, and consent in healthcare and research. Digit Heal. 2024;10:20552076241290964. 10.1177/20552076241290964 PMC11504247 39465223

[7] Murphy SN , Mendis M, Hackett K, et al Architecture of the open-source clinical research chart from informatics for integrating biology and the bedside. AMIA Annu Symp Proc. 2007;2007:548–552. 18693896 PMC2655844

[8] Wagholikar KB , Dessai P, Sanz J, et al Implementation of informatics for integrating biology and the bedside (i2b2) platform as docker containers. BMC Med Inform Decis Mak. 2018;18:66. 10.1186/s12911-018-0646-2 30012140 PMC6048900

[9] Murphy S , Wilcox A. Mission and sustainability of informatics for integrating biology and the bedside (i2b2). EGEMS (Wash DC). 2014;2:1074. 10.13063/2327-9214.1074 25848608 PMC4371505

[10] Wang X , Williams C, Liu ZH, Croghan J. Big data management challenges in health research-a literature review. Brief Bioinform. 2019;20:156–167. 10.1093/bib/bbx086 28968677 PMC6488939

[11] Waring J , Lindvall C, Umeton R. Automated machine learning: Review of the state-of-the-art and opportunities for healthcare. Artif Intell Med. 2020;104:101822. 10.1016/j.artmed.2020.101822 32499001

[12] Wagholikar KB , Ainsworth L, Zelle D, et al I2b2-etl: Python application for importing electronic health data into the informatics for integrating biology and the bedside platform. Bioinformatics. 2022;38:4833–4836. 10.1093/bioinformatics/btac595 36053173 PMC9563689

[13] Smith JW , Everhart JE, Dickson WC, Knowler WC, Johannes RS. Using the ADAP learning algorithm to forecast the onset of diabetes mellitus. Proc Annu Symp Comput Appl Med Care. 1988:261–265.

[14] Johnson AEW , Bulgarelli L, Shen L, et al MIMIC-IV, a freely accessible electronic health record dataset. Sci Data. 2023;10:219. 10.1038/s41597-022-01899-x 36596836 PMC9810617

[15] Kapoor S , Narayanan A. Leakage and the reproducibility crisis in machine-learning-based science. Patterns (N Y). 2023;4:100804. 10.1016/j.patter.2023.100804 37720327 PMC10499856

[16] Wagholikar KB , Ainsworth L, Vernekar VP, et al Extending i2b2 into a framework for semantic abstraction of EHR to facilitate rapid development and portability of health IT applications. AMIA Jt Summits Transl Sci Proc. 2019;2019:370–378. 31258990 PMC6568124

[17] Thayer DS , Mumtaz S, Elmessary MA, et al Creating a next-generation phenotype library: the health data research UK phenotype library. JAMIA Open. 2024;7:ooae049. 10.1093/jamiaopen/ooae049 38895652 PMC11182945

[18] Honerlaw J , Ho Y-L, Fontin F, et al Centralized interactive phenomics resource: an integrated online phenomics knowledgebase for health data users. J Am Med Inform Assoc. 2024;31:1126–1134. 10.1093/jamia/ocae042 38481028 PMC11031216

[19] Brandt PS , Pacheco JA, Rasmussen LV. Development of a repository of computable phenotype definitions using the clinical quality language. JAMIA Open. 2021;4:ooab094. 10.1093/jamiaopen/ooab094 34926996 PMC8672934

[20] Reps JM , Schuemie MJ, Suchard MA, Ryan PB, Rijnbeek PR. Design and implementation of a standardized framework to generate and evaluate patient-level prediction models using observational healthcare data. J Am Med Inform Assoc. 2018;25:969–975. 10.1093/jamia/ocy032 29718407 PMC6077830

