# Supplementary material for: i2b2-ML: module to facilitate machine learning in the informatics for integrating biology and the bedside platform
Source: JAMIA Open. 2026 May 25;9(3):ooag047. doi: 10.1093/jamiaopen/ooag047 (PMC13200738; doi:10.1093/jamiaopen/ooag047)
Supplement: ooag047_Supplementary_Data [file ooag047_supplementary_data.zip › appendix_b.pdf]

Appendix B

User workflow for building and executing ML models

For developing an ML model, the researcher first creates training data by creating a positive patient-set and a negative patient-set using the i2b2 query interface. The data for the patients in these patient sets constitute the training data for creation of the ML model. The positive patient set consists of patients that have the attribute or outcome to be predicted. In contrast the negative patient set is the control group of patients that do not have the attribute or outcome of interest. The workflow for ML involves the end-user using the query interface of i2b2 for defining patient-sets, and then using the Swagger interface which accepts JSON text to trigger the processes to build or execute the models. For instance, a researcher who wants to train an ML model (e.g., for finding diabetes patients) performs the following steps.

1. The researcher creates a query in the i2b2 interface, to find patients that have been annotated for the presence of diabetes (referred as the positive set) and similarly creates a negative-set query to find patients that have been annotated for absence of diabetes.
2. After the patient sets have been created, the researcher creates a concept for the ML model with specifications in the Swagger interface. The specification for creating a diabetes model is shown in figure 2a.
    a. Description. Human readable description for the model that will be shown as tooltip in the query interface.
    b. Path of Concept in the i2b2 ontology, where the model will be stored
    c. Names of positive and negative patient sets (from step 2)
    d. Concept code. The code that will be used to store facts output by the model.
    e. Data paths. The array of paths to the concepts (including their descendants) that will be used as features for training the model
    f. Label paths. The array of paths to the events (including their descendants) that will be used as labels for the positive set of patients.
    g. Random Seed. Number between 0-1 for seeding random number generator
    h. Data period. The start and end times that the training data will be restricted to.
    i. Time buffer. The period in seconds prior to the label event (gold-standard event or annotation) during which the data is excluded from the training set. The buffer period for data isolation is often necessary to avoid data leakage. For example, the diagnosis of cancer may be immediately preceded by a surgical biopsy to confirm cancer, and inclusion of this antecedent data point would reduce the utility of the model for screening patients.

3. The researcher then triggers a process to build the ML model indicating the path to the concept where the ML model was created in the previous step. The example specifications for the diabetes example are shown in figure 2b.

For executing the model, the researcher creates a query in the i2b2 interface, to build a 'target patient-set', on which the ML model will be executed on. This set could include patients that have a predisposing risk factor for the disease. Next the user proceeds to build the ML model in the Swagger interface by using the following specifications. See figure 2c for the diabetes example.

a. Concept-path of the model.
b. Name of patient set created.
c. Prediction-event path is the path to the concept at whose first occurrence the model prediction is to be made. The data in the test set is excluded on and after this point. Additionally, the time buffer is applied relative to this point.
d. Time buffer. The period in seconds prior to the prediction event during which the data is excluded from the feature set that will serve as input to the ML model.
e. Data period. The start and end times that the input data will be restricted to.

The internal workflows for building and executing the ML model are summarized in figures B1 and B2.
