# Supplementary material for: i2b2-ML: module to facilitate machine learning in the informatics for integrating biology and the bedside platform
Source: JAMIA Open. 2026 May 25;9(3):ooag047. doi: 10.1093/jamiaopen/ooag047 (PMC13200738; doi:10.1093/jamiaopen/ooag047)
Supplement: ooag047_Supplementary_Data [file ooag047_supplementary_data.zip › i2b2-ml-manuscript-v10-a.pdf]

Appendix A

Challenges associated with the development of machine learning models external to the i2b2 environment:

1. **Data Security and Privacy**: When downloading patient data, there are regulations and ethical considerations to ensure data privacy and security, even if the data is de-identified. Researchers must handle sensitive health information carefully, ensuring adherence to regulations like Health Insurance Portability and Accountability Act (HIPPA).[10] Transferring data from a secure environment like i2b2 to local systems can increase the risk of accidental disclosures or unauthorized access.
2. **Data Management**: Managing large volumes of data locally requires significant data management effort for data storage, backup, and efficient retrieval.[11]
3. **Computational Resources**: Developing ML models requires substantial computational infrastructure, which may not be readily available to all research teams, and setting up a local ML development environment is often not cost-effective.
4. **Data science expertise**: ML development requires expertise in data science, statistics, and programming. Researchers must be proficient in using ML tools and frameworks in Python and R. The need to switch between different tools and environments (from i2b2 for data extraction to local environments for ML development) can add complexity and require broader technical skills.[12]
5. **Reproducibility and Validation**: Ensuring the reproducibility of ML models can be challenging. Variability in data preprocessing, ML development frameworks, and evaluation metrics can lead to results that are difficult to replicate. This is particularly critical in medical research, where reproducibility is essential for validating study findings and ensuring their applicability in clinical settings.[2]
6. **Model Deployment and Integration**: Once developed, integrating the output of the ML models back into i2b2 platform for supporting downstream analytics poses additional challenges. Additionally, the need for model updating and maintenance, and ensuring the model operates reliably within the study period are significant considerations.[2,12]
